# DNA double-strand breaks alter the spatial arrangement of homologous loci in plant cells

**DOI:** 10.1038/srep11058

**Published:** 2015-06-05

**Authors:** Takeshi Hirakawa, Yohei Katagiri, Tadashi Ando, Sachihiro Matsunaga

**Affiliations:** 1 Department of Applied Biological Science, Faculty of Science and Technology, Tokyo University of Science, 2641 Yamazaki, Noda, Chiba 278-8510, Japan; 2 Laboratory for Biomolecular Function Simulation, Computational Biology Research Core, RIKEN Quantitative Biology Center, International Medical Device Alliance, 1-6-5 Minatojima-minamimachi, Chuo-ku, Kobe, Hyogo 650-0074, Japan

## Abstract

Chromatin dynamics and arrangement are involved in many biological processes in nuclei of eukaryotes including plants. Plants have to respond rapidly to various environmental stimuli to achieve growth and development because they cannot move. It is assumed that the alteration of chromatin dynamics and arrangement support the response to these stimuli; however, there is little information in plants. In this study, we investigated the chromatin dynamics and arrangement with DNA damage in *Arabidopsis thaliana* by live-cell imaging with the *lacO*/LacI-EGFP system and simulation analysis. It was revealed that homologous loci kept a constant distance in nuclei of *A. thaliana* roots in general growth. We also found that DNA double-strand breaks (DSBs) induce the approach of the homologous loci with γ-irradiation. Furthermore, AtRAD54, which performs an important role in the homologous recombination repair pathway, was involved in the pairing of homologous loci with γ-irradiation. These results suggest that homologous loci approach each other to repair DSBs, and AtRAD54 mediates these phenomena.

Chromatin structure, dynamics, and arrangement in the nucleus are closely related to many biological processes such as DNA replication, transcription, and repair in eukaryotes including plants^1^,^2^,^3^. In nuclei of *Arabidopsis thaliana*, several chromatins form territories called chromosome territories (CTs)^4^. CT arrangement is regulated by nuclear proteins, and it appears to be important for growth and fertility in *A. thaliana*^5^. In recent years, three-dimensional chromatin structure in *A. thaliana* was revealed by the Hi-C method, and it is different from that of humans and *Drosophila melanogaster*^6^,^7^. Chromatin structure was related to epigenetic modification in those studies; therefore, it is also important for gene regulation and DNA replication in *A. thaliana* as well as other organisms. There are similar findings about chromatin arrangement and structure as above; however, little is known about chromatin dynamics in nuclei of *A. thaliana*. Fluorescence *in situ* hybridisation (FISH) and immunofluorescence staining has been mainly used to analyse the gene localization and chromatin distribution in nuclei of *A. thaliana*. These attractive methods allow the observation of chromatin arrangement in various tissues without requiring the production of transgenic lines; however, they require fixation of cells and high-temperature treatment for hybridisation. Therefore, they cannot be adapted to *in vivo* detection of chromatin dynamics and arrangement. Moreover, it is difficult to perform these methods in tissues and organs while maintaining their morphology. Recent advances in live-cell imaging techniques reveal the movement of genomic loci *in vivo* in DNA replication^8^, transcription^9^, and repair^10^. One live-cell imaging technique, a chromatin-tagging system that is based on the bacterial operator/repressor system, has been a powerful technique for analysing chromatin dynamics and arrangement in real time^11^,^12^. Plants have to respond rapidly to DNA damage, which threatens the genome stability that is required for correct growth and development, because they cannot move^13^,^14^. It is assumed that alterations of chromatin dynamics support the response to DNA damage; however, little is known in plants. Here, we examined chromatin dynamics and arrangement in living roots of *A. thaliana* with DNA damage, focusing our attention on the distance between homologous loci using the *lacO*/LacI-EGFP system. We revealed that the homologous loci kept a constant three-dimensional distance in the nucleus using live-cell imaging with a bacterial operator/repressor system. Moreover, the distance between two homologous loci in the interphase nucleus was shortened by γ-irradiation, which induces DNA double-strand breaks (DSBs). We found that AtRAD54, which performs an important role in the homologous recombination (HR) repair pathway, was involved in the approach of two homologous loci under γ-irradiation. Our results suggest that the transient reduction in inter-allelic distance and increase in pairing frequency of homologous loci after DSB result in partial chromatin reorganisation of interphase nuclei and that AtRAD54 contributes to the subcellular movement of homologous loci in the HR repair pathway.

## Results

### Distance of homologous loci is constant in nuclei of *A. thaliana*

**Figure 1:**
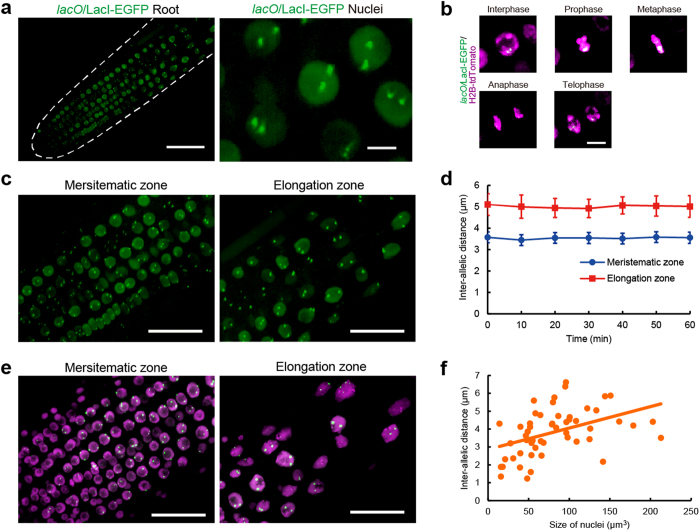
Visualisation of homologous loci with *lacO*/LacI-EGFP in *Arabidopsis thaliana* roots. **a,** Root (left, scale bar = 50 μm) and nucleus (right, scale bar = 5 μm) in *A. thaliana* expressing *lacO*/LacI-EGFP. The white dotted line shows the appearance of a root. **b,** *lacO*/LacI-EGFP signals during mitosis in *A. thaliana* expressing *lacO*/LacI-EGFP in green and H2B-tdTomato in magenta. Scale bar = 5 μm. **c,** Nuclei in the meristematic zone and elongation zone of *A. thaliana* expressing *lacO*/LacI-EGFP. Scale bars = 30 μm. **d,** Dynamics of the inter-allelic distances in the meristematic zone and elongation zone nuclei (*n* = 20, time interval = 10 min). **e,** Nuclei in the meristematic zone and elongation zone of *A. thaliana* expressing *lacO*/LacI-EGFP and H2B-tdTomato. Scale bars = 30 μm. **f**, Correlation between the inter-allelic distance and the size of the nucleus in *A. thaliana* roots. The correlation coefficient was 0.44 (*n* = 53, **P < 0.01).

**Figure 2:**
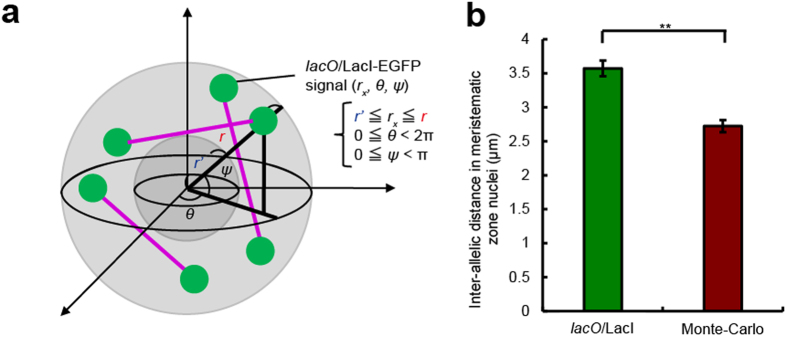
Simulation analysis of the distance between homologous loci using a mathematical model. **a,** Mathematical model mimicking a nucleus in the meristematic zone of *A. thaliana* roots. This model simulated random inter-allelic distances (drawn in purple lines) in the nucleoplasm and calculated their average. **b,** Comparison between the measured value (*lacO*/LacI) and the simulated value (Monte Carlo) (*n* = 105, **P < 0.01).

In root tips of *A. thaliana* expressing *lacO*/LacI-EGFP, two dots of EGFP signal derived from homologous loci were detected in each nucleus (Fig. 1a). To observe the dynamics and arrangement of *lacO*/LacI-EGFP signals during mitosis, we generated a transgenic line of *A. thaliana* stably expressing both *lacO*/LacI-EGFP and H2B-tdTomato. In the meristematic zone of roots, the alignment of *lacO*/LacI-EGFP signals was observed on mitotic chromosomes at the metaphase plate (Fig. 1b, Supplementary Video 1). We focused on the distance between homologous loci, which is called the inter-allelic distance. To analyse the spatiotemporal dynamics of the inter-allelic distance, we performed time-lapse imaging of root nuclei in *A. thaliana* expressing *lacO*/LacI-EGFP. When the three-dimensional distance was measured, it was nearly constant in nuclei of both meristematic and elongation zones, although the nuclear morphology drastically changed. The inter-allelic distance in nuclei of the elongation zone was longer than that in nuclei of the meristematic zone (Fig. 1c,d, Supplementary Video 2, 3). In the elongation zone of *A. thaliana*, multiple DNA replications, which are known as endoreplications and endocycles, result in the formation of larger nuclei than those in the meristematic zone^15^. Then, to examine whether the inter-allelic distance depends on the size of the nucleus, we measured the inter-allelic distance and the volume of the same nuclei in roots of *A. thaliana* expressing *lacO*/LacI-EGFP and H2B-tdTomato. A correlation between the inter-allelic distance and the size of nucleus was found in roots (Fig. 1e,f). These results indicate that the inter-allelic distance depends on the size of the nucleus in *A. thaliana* roots. To investigate whether the inter-allelic distance is stochastically determined, we developed a mathematical model that mimics the nucleus in the meristematic zone (Fig. 2a). In this model, we calculated distances between two randomly generated points in a nucleoplasm in a Monte Carlo procedure^16^. The average value of inter-allelic distances measured in nuclei of the meristematic zone was significantly longer than the value obtained by the mathematical model (Fig. 2b). This result also suggests that the inter-allelic distance is constant in nuclei of *A. thaliana* roots.

### DSBs induce the approach of homologous loci

**Figure 3:**
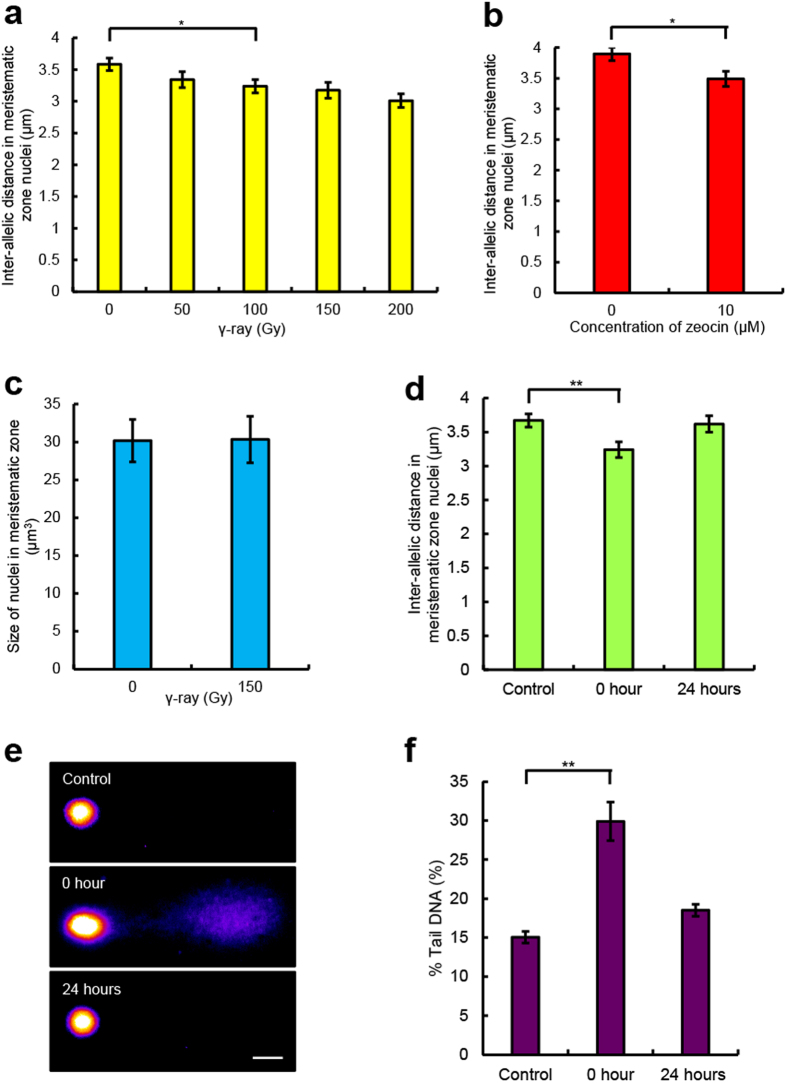
Effect of DNA double-strand breaks (DSBs) on the inter-allelic distance. **a**,**b,** Relationship between the inter-allelic distance and dose of γ-irradiation (*n* > 90, *P < 0.05, **a**) or 10 μM zeocin (*n* > 90, *P < 0.05, **b,**) in the meristematic zone of *A. thaliana* roots. **c,** Size of nuclei in the meristematic zone of *A. thaliana* roots irradiated with 150 Gy γ-irradiation (*n* = 122, *P* = 0.35). **d,** Inter-allelic distance in the meristematic zone nuclei of *A. thaliana* irradiated with 150 Gy γ-irradiation (*n* ≥ 90, **P < 0.01). **e,** Images of comets representing the control nucleus and the nucleus irradiated with 150 Gy γ-irradiation (top, Control; middle, 0 h; bottom, 24 h). Scale bar = 10 μm. **f,** Levels of DSBs that were detected by a comet assay in *A. thaliana* roots irradiated with 100 Gy γ-irradiation (*n* ≥ 70, **P < 0.01).

DNA damage induces chromatin rearrangement in nuclei of human cells^17^. Therefore, we anticipated that the inter-allelic distance would be changed by DNA damage, and we focused on the inter-allelic distance with γ-irradiation and the radiomimetic reagent zeocin, which induce DSBs^18^. We measured inter-allelic distances in interphase nuclei after γ-irradiation and zeocin treatment. When the plant was irradiated with more than 100 Gy γ-irradiation or treated with 10 μM zeocin, the inter-allelic distance was significantly shortened (Fig. 3a,b). The distance became shorter as the dose of γ-irradiation was increased. This reduction of the inter-allelic distance in a dose-dependent manner should reflect the amount of DSBs after γ-irradiation. To investigate whether the alteration of the inter-allelic distance by γ-irradiation depends on the secondary effect of the increase in the size of the nucleus, we measured the nucleus size after γ-irradiation. In the meristematic zone, the nucleus volume after γ-irradiation was not significantly different from that before γ-irradiation (Fig. 3c). Therefore, these results demonstrate that the reduction in inter-allelic distance is directly caused by γ-irradiation and is not accompanied with DNA replication. Next, because we anticipated that the close approach of homologous loci was important for DNA repair, we examined the recovery of the inter-allelic distance in a time-course experiment after γ-irradiation. The shortened inter-allelic distance at 0 h after γ-irradiation was recovered to the original distance at 24 h after γ-irradiation (Fig. 3d). We also investigated the level of DSBs using a comet assay^19^. The level of DSBs was recovered at 24 h after γ-irradiation (Fig. 3e,f). In nuclei of human cells, chromatin arrangement, which is altered by DNA damage, returns after DNA repair^17^. These results suggest that homologous loci approach each other for DNA repair with γ-irradiation.

### AtRAD54 mediates the pairing of homologous loci with DSBs

**Figure 4:**
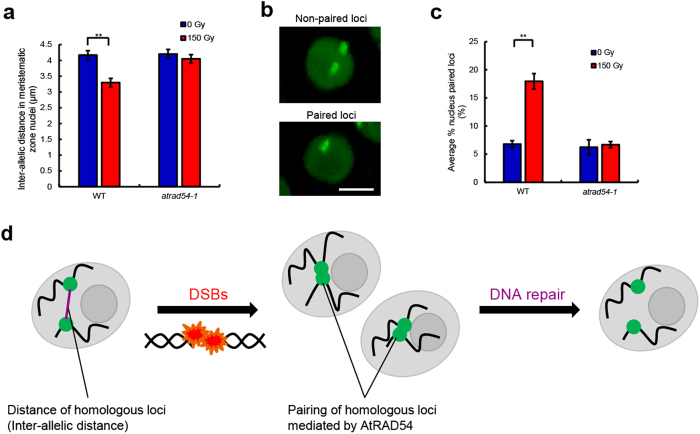
Regulation of the inter-allelic distance by AtRAD54 with DSBs. **a,** Inter-allelic distance in meristematic zone nuclei of wild-type and *atrad54-1* roots irradiated with 150 Gy γ-irradiation (*n* > 90, **P < 0.01). **b,** Images of nuclei with paired loci and non-paired loci in the meristematic zone. Scale bar = 5 μm. **c,** Frequency of meristematic zone nuclei with paired homologous loci in wild-type and *atrad54-1* roots irradiated with 150 Gy γ-irradiation. Five roots containing at least 30 nuclei were counted for each group (**P < 0.01). **d,** Diagram of this research showing that the distance between homologous loci was constant and that DSBs induced the frequency of homologous loci pairing.

When DNA damage including DSBs occurs in cells, DNA repair factors are expressed and repair damage in nuclei of *A. thaliana*^13^,^14^. Therefore, we expected that DNA repair factors were involved in the event that the inter-allelic distance was shortened by DSBs. There are at least four DSB repair pathways including the canonical-non homologous end joining (C-NHEJ), alternative-NHEJ (A-NHEJ), microhomology-mediated EJ (MMEJ), and HR repair pathway in *A. thaliana*^20^. We generated *atlig4-4* and *atrad54-1* plants expressing *lacO*/LacI-EGFP. AtLIG4 has ATP-dependent DNA ligase activity and catalyses the final step in the C-NHEJ pathway^21^. In contrast, AtRAD54, which is a chromatin re-modelling factor belonging to the SWI2/SNF2 family, has an important role in the HR repair pathway^22^. Although the inter-allelic distance shortened after γ-irradiation in the wild-type (Col-0) and *atlig4-4* cells, it was not significantly shortened in *atrad54-1* cells (Fig. 4a, Supplementary Fig. 1a). We observed the overlapped foci of two *lacO*/LacI-EGFP signals at low frequency (<7%), suggesting that these homologous loci were paired (Fig. 4b). When we measured the inter-allelic distance, the overlapped loci were excluded from the calculation. Although the frequency was not changed between wild-type and mutant cells before γ-irradiation, the frequency after γ-irradiation in *atrad54-1* was lower than that of wild-type and *atlig4-4* cells (Fig. 4b,c, Supplementary Fig. 1b). These results suggest that homologous loci might approach each other to repair DSBs through HR.

## Discussion

We used the *lacO*/LacI-EGFP system, which allowed us to visualise specific loci where the tandem operator array was inserted in this study. Chromatin regions tagged with repetitive *lacO* sequences tend to form pairs with each other more frequently than regions without the sequences^2^,^23^. However, we could measure the constant values of inter-allelic distance in interphase nuclei of both meristematic and elongation regions using line 25:26, in which the pairing of *lacO* arrays was not higher than that of control loci^23^,^24^. Live-cell imaging and simulation analysis revealed that the inter-allelic distance is constant in interphase nuclei of *A. thaliana* roots. The inter-allelic distance of nuclei in the meristematic region of *A. thaliana* is approximately 3.5 μm, whereas that of *Saccharomyces cerevisiae* and SG4 cultured cells of *D. melanogaster* is about 1.2 μm and 2 μm, respectively^10^,^25^. In plants and other organisms, the arrangement of homologous loci is regulated by various factors in interphase nuclei. Homologous loci are dispersed by condensin II, which is a protein complex that contributes to chromosome condensation and segregation during mitosis, in *D. melanogaster*^25^. Condensin II also separates sister chromatids during S phase in human cells^26^. Sister chromatids are separated in nuclei of *A. thaliana* leaves by the SMC5/6 complex, which is required for chromosome segregation during mitosis^27^. In *S. cerevisiae*, the inter-allelic distance is constant; however, the mechanism that regulates the inter-allelic distance is unknown^10^. Our results suggest that there are sub-nuclear mechanisms that separate homologous chromosomes during interphase in *A. thaliana*. When DSBs occur in nuclei of *A. thaliana* roots, the inter-allelic distance shortened. These results might suggest that homologous loci approached each other to repair DSB loci through HR. In *S. cerevisiae*, RAD54, which is a multifunctional factor for RAD51-mediated HR^28^, catalyses nucleosome redistribution to permit the pairing of homologous loci in synapsis^29^,^30^ and contributes to the long-range search for homologous loci after a DSB occurs^31^. Furthermore, RAD54 belongs to the epistasis protein family of RAD52^32^, which has several activities in HR^10^. AtRAD54 interacts with AtRAD51, and *atrad54* reduces the frequency of HR in *A. thaliana*^22^. In *atrad54-1* cells expressing *lacO*/LacI-EGFP, the distance between homologous loci was not shortened, and the frequency of nuclei with paired loci did not increase after γ-irradiation. These results might suggest that the approach of homologous loci is mediated by RAD54 after γ-irradiation (Fig. 4c,d). Pairing of homologous loci for DNA repair started 90 min and peaked 2 h after a DSB event in *S. cerevisiae*^10^. Because γ-irradiation to *A. thaliana* took 3 h, our observed reduction in inter-allelic distance after DSB could exhibit the following situation after pairing with homologous loci. In this study, we cannot exclude the possibility that the approach of homologous loci occurs because of the collapse of all chromosomes with DSBs. To resolve this problem, it would be effective to investigate the distance between non-homologous loci with DSBs. However, measurements of the distance between non-homologous loci based on the bacterial operator/repressor system are difficult because the simultaneous expression of different fluorescent proteins frequently induces silencing^24^. Visualisation of specific loci with genome editing^33^,^34^ in cultured animal cells might be applicable to investigate the behaviour of non-homologous loci in nuclei. DNA damage induces not only the pairing of homologous loci but also chromatin rearrangements in *S. cerevisiae* and humans^10^,^17^. Our results suggest that the transient reduction in inter-allelic distance and increase in pairing of homologous loci after DSBs might be accompanied by a partial modification of chromatin organisation through the local CT perturbation. Further studies to reveal the relationship between the movement of damaged loci and spatial alteration of the CT would lead us to a more detailed understanding of the mechanism of chromatin organisation during the DNA repair process.

## Methods

### Plant materials and growth conditions

*A. thaliana* (Col-0 accession) expressing *lacO*/LacI-EGFP was kindly provided by Antonius J. M. Matzke (Institute of Plant and Microbial Biology), *atlig4-4* (Col-0 accession) was kindly provided by Toru Fujiwara (The University of Tokyo), and *atrad54-1* (Col-0 accession) was kindly provided by Keishi Osakabe (The University of Tokushima). Seeds of *A. thaliana* were germinated on Murashige and Skoog (MS) medium plates (1/2 MS salts, 1% [w/v] sucrose, 1.5% [w/v] gellan gum). Plates were placed at 4 °C for 1 day and then moved to an incubator and grown at 22 °C in a 16-h light/8-h dark cycle.

### Time-lapse imaging and three-dimensional analysis

Seeds of *A. thaliana* expressing *lacO*/LacI-EGFP were germinated on MS medium in a glass-bottomed dish. The dish was placed at 4 °C for 1 day and then moved to an incubator and grown at 22 °C in a 16-h light/8-h dark cycle. Seedling roots were observed 5 days after germination under an inverted fluorescent microscope (IX-81, Olympus) equipped with a confocal scanning unit (CSU-X1, Yokogawa) and an sCMOS camera (Neo 5.5 sCMOS, ANDOR). One stack of 0.5-μm z-axis steps was collected for 60 min (time interval: 10 min). Images were analysed with an ImageJ software plugin LP StackLine from LPixel (http://lpixel.net/). The meristematic zone and elongation zone were distinguished by the distance of adjacent nuclei as previously described^15^.

### γ-irradiation and chemical treatment

Five days after germination, seedlings were γ-irradiated using a^137^Cs source (Research Institute for Biomedical Sciences, Tokyo University of Science) at a dose rate of 0.83 Gy/min. Five days after germination, seedlings were transferred to MS medium containing zeocin (Invitrogen). They were incubated for 1 day at 22 °C in a 16-h light/8-h dark cycle.

### Comet assay

A comet assay was performed with a neutral electrophoresis without alkaline denaturation protocol as previously described^19^. Comet slides were not stained with ethidium bromide but with SYBR Green I (Invitrogen). Images of comets stained with SYBR Green I were acquired under an upright microscope equipped with a CCD camera (Cool Snap HQ2, Nippon Roper). Images were analysed with an ImageJ software plugin Comet Assay from Microscopy Services Laboratory (https://www.med.unc.edu/microscopy).

### Monte Carlo simulation

We calculated distances between two randomly generated points in a spherical shell that satisfy *r′* < *r*_*x*_ ≤ *r* whe*r*e *r*' is the radius of the nucleolus (2.3 μm), *r* is the radius of the nucleus (1.17 μm), and *r*_*x*_ is the distance of point *x* (*x* = 1 and 2) from the origin.

## Additional Information

**How to cite this article**: Hirakawa, T. *et al.* DNA double-strand breaks alter the spatial arrangement of homologous loci in plant cells. *Sci. Rep.* **5**, 11058; doi: 10.1038/srep11058 (2015).

## Supplementary Material

Supplementary Information

Supplementary video 1

Supplementary video 2

Supplementary video 3
