# Supplementary material for: DNA double-strand breaks alter the spatial arrangement of homologous loci in plant cells
Source: Sci Rep. 2015 Jun 5;5:11058. doi: 10.1038/srep11058 (PMC4457028; doi:10.1038/srep11058)
Supplement: Supplementary Information [file srep11058-s1.pdf]

**Supplementary information**

# DNA double-strand breaks alter the spatial arrangement of homologous loci in plant cells

Takeshi Hirakawa[1], Yohei Katagiri[1], Tadashi Ando[2], and Sachihiro Matsunaga[1*]

[1]Department of Applied Biological Science, Faculty of Science and Technology, Tokyo University of Science, 2641 Yamazaki, Noda, Chiba 278-8510, Japan

[2]Laboratory for Biomolecular Function Simulation, Computational Biology Research Core, RIKEN Quantitative Biology Center, International Medical Device Alliance, 1-6-5 Minatojima-minamimachi, Chuo-ku, Kobe, Hyogo 650-0074, Japan

[*]Corresponding author

Department of Applied Biological Science, Faculty of Science and Technology, Tokyo University of Science, 2641 Yamazaki, Noda, Chiba 278-8510, Japan

Tel: +81-4-7124-1501

Fax: +81-4-7123-9767

Email: sachi@rs.tus.ac.jp

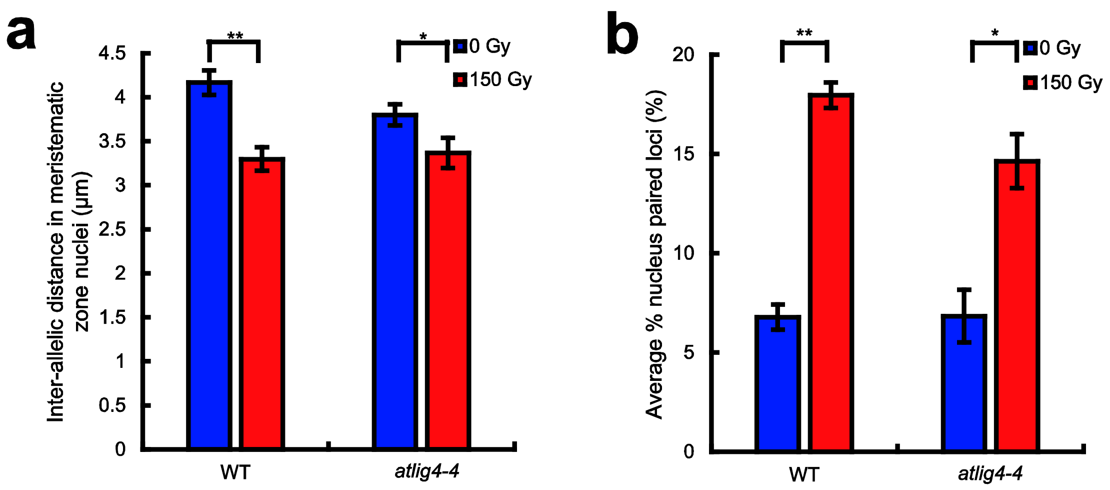


**Supplementary Figure 1 Effect of DNA double-strand breaks on the inter-allelic distance in *atlig4-4* roots.**

**a,** Inter-allelic distance in meristematic zone nuclei of wild-type and *atlig4-4* roots irradiated with 150 Gy γ-irradiation ($n > 90$, *$P < 0.05$, **$P < 0.01$). **b,** Frequency of meristematic zone nuclei with paired homologous loci in wild-type and *atlig4-4* roots irradiated with 150 Gy γ-irradiation. Five roots containing at least 30 nuclei were counted for each group (*$P < 0.05$, **$P < 0.01$).

**Supplementary video 1**

Dynamics of homologous loci during the cell cycle in a meristematic zone nucleus of an *Arabidopsis thaliana* root expressing *lacO*/LacI-EGFP and H2B-tdTomato.

**Supplementary video 2**

Dynamics of homologous loci during interphase in a meristematic zone nucleus of an *A. thaliana* root expressing *lacO*/LacI-EGFP.

**Supplementary video 3**

Dynamics of homologous loci during interphase in an elongation zone nucleus of an *A. thaliana* root expressing *lacO*/LacI-EGFP.
